# Efficacy and safety of iruplinalkib (WX-0593) in *ALK*-positive crizotinib-resistant advanced non-small cell lung cancer patients: a single-arm, multicenter phase II study (INTELLECT)

**DOI:** 10.1186/s12916-023-02738-5

**Published:** 2023-02-24

**Authors:** Yuankai Shi, Jianhua Chen, Helong Zhang, Zhihong Zhang, Yiping Zhang, Zhehai Wang, Shucai Zhang, Jian Zhao, Chunling Liu, Xiuwen Wang, Yanqiu Zhao, Changlu Hu, Lei Yang, Xuezhi Hao, Lin Wang, Yunpeng Liu, Yan Yu, Jun Zhao, Mengzhao Wang, Liangming Zhang, Sanyuan Sun, Yanping Hu, Kangsheng Gu, Xiaosheng Hang, Jinlu Shan, Yu Zhang, Bangxian Tan, Weihua Yang, Runxiang Yang, Meimei Si, Huaize Geng, Hui Li, Xiaoyan Kang

**Affiliations:** 1 Department of Medical Oncology, National Cancer Center/National Clinical Research Center for Cancer/Cancer Hospital, Chinese Academy of Medical Sciences & Peking Union Medical College, Beijing Key Laboratory of Clinical Study on Anticancer Molecular Targeted Drugs, Beijing, China; 2 Thoracic Medicine Department I, Hunan Tumor Hospital, Changsha, China; 3 Oncology Department, the Second Affiliated Hospital of Air Force Medical University, Xi’an, China; 4 Department of Respiratory Oncology, Anhui Provincial Cancer Hospital, Hefei, China; 5 Thoracic Medical Oncology, Zhejiang Cancer Hospital, Hangzhou, China; 6 Respiratory Medical Oncology Ward II, Shandong Provincial Institute of Cancer Prevention and Treatment, Jinan, China; 7 Oncology Department II, Beijing Chest Hospital, Capital Medical University, Beijing, China; 8 Thoracic Surgery I, Affiliated Cancer Hospital and Institute of Guangzhou Medical University, Guangzhou, China; 9 Pulmonary Medicine Ward II, The Affiliated Tumour Hospital of Xingjiang Medical University, Urumqi, China; 10 Chemotherapy Department, Qilu Hospital of Shandong University, Jinan, China; 11 Respiratory Medicine Ward I, The Affiliated Cancer Hospital of Zhengzhou University & Henan Cancer Hospital, Zhengzhou, China; 12 Ward IV of Department of Oncology, Anhui Provincial Cancer Hospital, Hefei, China; 13 Department of Respiratory Oncology, Gansu Province Cancer Hospital, Lanzhou, China; 14 Department of Medical Oncology Ward II, The First Hospital of China Medical University, Shenyang, China; 15 Respiratory Medicine Ward III, Harbin Medical University Cancer Hospital, Harbin, China; 16 Department of Thoracic Oncology I, Peking University Cancer Hospital, Beijing, China; 17 Department of Pulmonary and Critical care Medicine, Peking Union Medical College Hospital, Chinese Academy of Medical Sciences & Peking Union Medical College, Beijing, China; 18 Oncology Department, Yantai Yuhuangding Hospital, Yantai, China; 19 Department of Medical Oncology, Xuzhou Central Hospital, Xuzhou, China; 20 Thoracic Oncology (2), Hubei Cancer Hospital, Wuhan, China; 21 Department of Oncology, the First Affiliated Hospital of Anhui Medical University, Hefei, China; 22 Medical Oncology, Affiliated Hospital of Jiangnan University, Wuxi, China; 23 Oncology Department, Army Medical Center of PLA, Chongqing, China; 24 Respiratory Department, Nanjing Chest Hospital, Medical School of Southeast University, Nanjing, China; 25 Department of Oncology, Affiliated Hospital of North Sichuan Medical college, Nanchong, China; 26 Department of Respiratory, Shanxi Provincial Cancer Hospital, Taiyuan, China; 27 The Second Department of Medical Oncology, Yunnan Cancer Hospital, Kunming, China; 28 Clinical Research Center, Qilu Pharmaceutical Co., Ltd., Jinan, China

**Keywords:** Iruplinalkib, WX-0593, Non-small cell lung cancer, Anaplastic lymphoma kinase, Tyrosine kinase inhibitor

## Abstract

**Background:**

Iruplinalkib (WX-0593) is an anaplastic lymphoma kinase (ALK)/c-ros oncogene 1 (ROS1) tyrosine kinase inhibitor. Here we reported the single-arm, phase II study (INTELLECT) results of the efficacy and safety of iruplinalkib for *ALK*-positive crizotinib-resistant advanced non-small cell lung cancer (NSCLC) patients.

**Methods:**

*ALK*-positive crizotinib-resistant advanced NSCLC patients aged ≥18 years, with Eastern Cooperative Oncology Group performance status of 0–2 were eligible. Patients received iruplinalkib 180 mg orally once daily for a 21-day cycle with a 7-day lead-in phase at 60 mg orally once daily. The primary endpoint was the independent review committee (IRC)-assessed objective response rate (ORR).

**Results:**

From August 7, 2019, to October 30, 2020, 146 patients were included. As of the data cut-off date on November 30, 2021, the median follow-up time was 18.2 months (95% confidence interval [CI] 16.8–18.8). IRC-assessed ORR and disease control rate (DCR) were 69.9% (95% CI 61.7–77.2%) and 96.6% (95% CI 92.2–98.9%), respectively. Investigator-assessed ORR and DCR were 63.0% (95% CI 54.6–70.8%) and 94.5% (95% CI 89.5–97.6%), respectively. Investigator-assessed median duration of response and progression-free survival (the same as median time to progression) were 13.2 months (95% CI 10.4–17.7) and 14.5 months (95% CI 11.7–20.0), respectively. Corresponding IRC-assessed results were 14.4 months (95% CI 13.1–not evaluable [NE]), 19.8 months (95% CI 14.5–NE), and NE (95% CI 14.5–NE), respectively. Investigator-assessed intracranial ORRs were 46% (41/90, 95% CI 35–56%) in patients with central nervous system metastases and 64% (27/42, 95% CI 48–78%) in patients with measurable intracranial lesions. Overall survival data were immature. Treatment-related adverse events (TRAEs) occurred in 136/146 (93.2%) patients. The most common TRAEs were aspartate aminotransferase increased (63 [43.2%]), alanine aminotransferase increased (54 [37.0%]), and blood creatine phosphokinase increased (51 [34.9%]). Dose interruption, reduction, and discontinuation due to TRAEs occurred in 21 (14.4%), 16 (11.0%), and four (2.7%) patients, respectively.

**Conclusions:**

In this study, iruplinalkib (WX-0593) demonstrated favorable efficacy and manageable safety profiles in patients with *ALK*-positive crizotinib-resistant advanced NSCLC. Iruplinalkib could be a new treatment option for this patient population.

**Trial registration:**

Center for Drug Evaluation of National Medical Products Administration of China: CTR20190789, registered on April 28, 2019; ClinicalTrials.gov: NCT04641754, registered on November 24, 2020.

**Supplementary Information:**

The online version contains supplementary material available at 10.1186/s12916-023-02738-5.

## Background

According to GLOBOCAN 2020, lung cancer incidence and mortality rate are far ahead worldwide [1]. Non-small cell lung cancer (NSCLC) accounts for approximately 80–85% of lung cancer [2]. Anaplastic lymphoma kinase (*ALK*) rearrangement occurred in 3–7% of NSCLC patients and has been proved to be an important carcinogenic driver [3–5]. The first-generation ALK tyrosine kinase inhibitor (TKI) crizotinib significantly improved the prognosis of patients with *ALK*-positive NSCLC [6]. However, most patients progressed within one year after crizotinib treatment. The main reason for drug resistance is insufficient penetration into the central nervous system (CNS) [7]. Second-generation ALK TKIs such as ceritinib, alectinib, brigatinib, ensartinib, and conteltinib (CT-707) have effectively addressed the issues of crizotinib resistance. For these novel ALK TKIs, objective response rates (ORR) were 33.3–56% and median progression-free survival (PFS) approximately ranged from six to 17 months in crizotinib-resistant disease [8–12]. Overcoming crizotinib resistance and developing new ALK TKIs are urgent clinical needs.

Iruplinalkib (WX-0593) is a novel and highly selective ALK/c-ros oncogene 1 (ROS1) TKI, which was developed by Qilu Pharmaceutical Co., Ltd., Jinan, China. The results of preclinical studies showed that it could effectively inhibit proliferation of crizotinib-sensitive or -resistant tumor cell lines, and *ALK*-positive crizotinib-sensitive or -resistant tumor growth in mouse models (data unpublished). The results of its phase I study showed that iruplinalkib had potent antitumor activity and manageable safety profile in crizotinib- or second-generation ALK TKI-resistant, or ALK TKI-naïve patients with *ALK*- or *ROS1*-positive advanced NSCLC [13]. For *ALK*-positive patients in the phase I study, ORRs were 63.0% (95% confidence interval [CI] 47.5–76.8%) in the dose-escalation phase and 58.2% (95% CI 47.4–68.5%) in the dose-expansion phase. Treatment-related adverse events (TRAEs) occurred in 92% of the patients at a dose of 180 mg orally once daily. It is noteworthy that lead-in phase had been implemented in the phase I study for safety concerns, and 180 mg orally once daily with a 7-day lead-in phase at 60 mg orally once daily was determined as the recommended phase 2 dose (RP2D) of iruplinalkib.

Here we reported the single-arm, phase II study (INTELLECT) results of the efficacy and safety of iruplinalkib for *ALK*-positive crizotinib-resistant advanced NSCLC patients.

## Methods

### Study design and participants

This study was conducted at 41 hospitals in China, in accordance with the Declaration of Helsinki and the International Council for Harmonisation guidelines for Good Clinical Practice. The study protocol was approved by the ethics committees of each participating hospital and registered on the Center for Drug Evaluation of National Medical Products Administration of China (CTR20190789) and ClinicalTrials.gov (NCT04641754). All patients provided written informed consent before participating in the study.

Eligible patients were ≥ 18 years old; with *ALK*-positive advanced NSCLC confirmed by histopathology or cytology; expected survival time ≥ 12 weeks; an Eastern Cooperative Oncology Group (ECOG) performance status (PS) of 0–2; progression after continuous treatment with crizotinib ≥ 12 weeks; ≥ one measurable extracranial lesion; and adequate organ function: absolute neutrophil count ≥ 1.5×10^9^/L, platelet count ≥ 90×10^9^/L, hemoglobin ≥ 90 g/L, total bilirubin ≤ 1.5×upper limit of normal (ULN) (for Gilbert’s syndrome: total bilirubin ≤ 3.0×ULN and direct bilirubin ≤ 1.5×ULN), alanine aminotransferase (ALT) and aspartate aminotransferase (AST) ≤ 2.5×ULN (for liver metastatic disease: ALT ≤ 5.0×ULN and AST ≤ 5.0×ULN), creatinine ≤ 1.5×ULN, and left ventricular ejection fraction ≥ 50%. Patients with asymptomatic brain metastases or those with symptomatic brain metastases who have been treated and stable for ≥ four weeks before iruplinalkib treatment initiation were allowed. Any surgery and radiotherapy (except palliative) must be completed ≥ four weeks before iruplinalkib treatment initiation. Palliative radiotherapy must be completed ≥ 48 h before iruplinalkib treatment initiation. *ALK*-alterations were tested before prior crizotinib treatment using immunohistochemical test (Ventana ALK [D5F3] CDx assay; Roche Tissue Diagnostics, Oro Valley, AZ, USA), amplification-refractory mutation system polymerase chain reaction (PCR), real-time PCR, fluorescence in situ hybridization, or next-generation sequencing.

Key exclusion criteria included previous ALK inhibitors other than crizotinib; continuous steroids > 30 days; planned to receive drugs that prolong QTc interval such as azithromycin or previously treated with those drugs within 14 days before iruplinalkib treatment initiation; or planned to receive strong inducer or inhibitor of CYP3A4 such as phenobarbital or previously treated with those drugs within seven days before iruplinalkib treatment initiation; with meningeal metastases; any clinically relevant cardiovascular or cerebrovascular disease such as myocardial infarction, heart failure within six months before iruplinalkib treatment initiation; or previous history of diffuse or bilateral lung interstitial fibrosis.

### Procedure

Patients received iruplinalkib 180 mg orally once daily for a 21-day cycle with a 7-day lead-in phase at 60 mg orally once daily until disease progression, intolerable toxicity, withdrawal of consent, loss of follow-up, start of other antitumor treatment, or death. If grade ≥ 2 cardiac or renal dysfunction or other grade ≥ 3 adverse events (AEs) occurred, iruplinalkib treatment would be suspended. At the discretion of investigator, iruplinalkib treatment would continue at the previous or a reduced dose after AEs reverted to grade ≤ 1 within 14 days. Two steps of iruplinalkib dose reduction to 120 mg and to 90 mg were allowed. If the same grade ≥ 3 AEs occurred again at a dose of 90 mg, treatment would discontinue. If dose suspension exceeded 14 cumulative days in a 21-day cycle or 14 continuous days, iruplinalkib dose discontinuation would also be required.

### Assessment

Efficacy examination was conducted within 28 days before iruplinalkib treatment initiation, every six weeks in the first 24 weeks after iruplinalkib treatment initiation, every nine weeks after 24 weeks, and every 12 weeks after one year until disease progression, withdrawal of consent, loss of follow-up, start of other antitumor treatment, or death, using computed tomography (CT) or magnetic resonance imaging according to the Response Evaluation Criteria in Solid Tumors (RECIST) version 1.1. And the CNS efficacy was evaluated on the basis of the response assessment in neuro-oncology (RANO) brain metastases criteria [14]. The imaging method used for each patient was consistent throughout the study. After the termination of iruplinalkib treatment, survival follow-up was performed every 12 weeks. AEs were classified and graded according to the National Cancer Institute Common Terminology Criteria for Adverse Events (CTCAE) version 4.03 from iruplinalkib treatment initiation throughout the treatment period and 28 days after the end of treatment. Plasma samples were obtained before administration of iruplinalkib on Cycle 1 Day 1, Cycle 1 Day 7, Cycle 1 Day 21, Cycle 2 Day 21, and Cycle 4 Day 21. Drug concentration of iruplinalkib was tested using liquid chromatography-mass spectrometry for pharmacokinetics (PK) evaluation.

### Outcomes

The primary endpoint was the independent review committee (IRC)-assessed ORR. Secondary endpoints included investigator-assessed ORR, disease control rate (DCR), duration of response (DoR), PFS, time to progression (TTP), and intracranial ORR (iORR), overall survival (OS), safety, and PK parameter (minimum concentration at steady-state [C_min,ss_]). Additionally, IRC-assessed DCR, DoR, PFS, and TTP were post hoc exploratory endpoints.

ORR was defined as the proportion of patients with complete response (CR) or partial response (PR). DCR was defined as the proportion of patients with CR, PR, or stable disease (SD). DoR was defined as the time from the date of the first CR or PR to the first time of disease progression or death from any cause, whichever occurred first. PFS was defined as the time from enrollment to the first time of disease progression or death from any cause, whichever occurred first, and TTP was defined as the time from enrollment to disease progression. iORR was defined as the proportion of patients with investigator-assessed intracranial CR or PR according to RANO brain metastases criteria. OS was defined as the time from enrollment to death from any cause.

The efficacy endpoints were evaluated in the full analysis set (FAS). The per-protocol set (PPS) and response evaluable set (RES) were used for sensitivity analyses. The FAS included all patients who received at least one dose of iruplinalkib. The PPS included patients in the FAS who complied with the protocol. The RES included patients in the FAS with measurable lesions by IRC at baseline. The safety analysis was conducted in the safety set (SS) which included patients who received at least one dose of iruplinalkib and one post baseline safety evaluation. PK were analyzed in patients who received at least one dose of iruplinalkib and provided at least one blood sample for the measurement of iruplinalkib concentrations.

### Statistical analysis

An ORR ≥ 40% was considered clinically acceptable and the expected ORR was 55%. One hundred and twenty-two patients were required to achieve a lower limit of the two-sided 95% CI of 40%, with a one-sided α of 0.025 and a power of 80%. Considering a dropout rate of 15%, a total sample size of 144 patients was required.

95% CIs for ORR and DCR were calculated using the Clopper-Pearson method. The median DoR, median PFS, median TTP, median OS, and corresponding 95% CIs were calculated using the Kaplan-Meier method. C_min,ss_ were summarized using descriptive statistics. All statistical analyses were performed by SAS (version 9.4, SAS Institute Inc., Cary, NC, USA).

## Results

### Patients

**Fig. 1:**
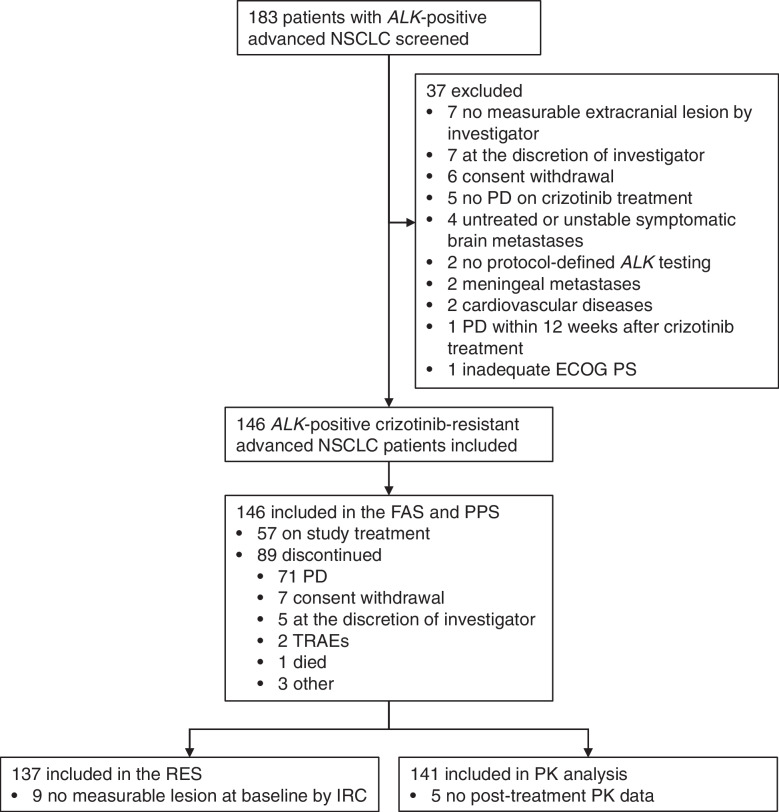
Study flow chart. *ALK*, anaplastic lymphoma kinase. NSCLC, non-small cell lung cancer. PD, progressive disease. ECOG, Eastern Cooperative Oncology Group. PS, performance status. FAS, full analysis set. PPS, per-protocol set. TRAEs, treatment-related adverse events. RES, response evaluable set. IRC, independent review committee. PK, pharmacokinetics

From August 7, 2019, to October 30, 2020, a total of 183 patients with *ALK*-positive advanced NSCLC were screened. One hundred and forty-six *ALK*-positive crizotinib-resistant advanced NSCLC patients were enrolled in this study, all of whom were included in the FAS and the PPS. The flow chart of this study is presented in Fig. 1. Nine patients had no measurable lesion at baseline by IRC, thus, the RES included 137 eligible patients. In the FAS, the mean age (± standard deviation) of patients was 52.4 ± 10.4 years old. Seventy-seven (52.7%) patients were female. Forty-two (28.8%) patients had ECOG PS of 0, and 99 (67.8%) had ECOG PS of 1. Brain metastases were found in 90 (61.6%) patients, of which 20 (22%) had received brain radiotherapy and 42 (47%) had measurable intracranial lesions. There were 56 (38.4%) patients with prior chemotherapy. One (0.7%) and 89 (61.0%) patients had CR and PR to prior crizotinib treatment, respectively (Table 1). All patients progressed on crizotinib treatment before the iruplinalkib treatment initiation. Comorbidities in > 5% patients were summarized in Additional file 1: Table S1. As of the data cut-off date on November 30, 2021, 57 (39.0%) patients were still on iruplinalkib treatment, and the median follow-up time was 18.2 months (95% CI 16.8–18.8).

**Table 1:** Patient baseline characteristics in FAS

Characteristics	Patients (n = 146)
Age, years (mean ± standard deviation)	52.4 ± 10.4
<65	127 (87.0%)
≥65	19 (13.0%)
Sex	
Male	69 (47.3%)
Female	77 (52.7%)
Ethnicity	
Han	144 (98.6%)
Other	2 (1.4%)
ECOG PS	
0	42 (28.8%)
1	99 (67.8%)
2	5 (3.4%)
Histopathologic type	
Adenocarcinoma	144 (98.6%)
Adenosquamous carcinoma	2 (1.4%)
Clinical stage	
IIIB	5 (3.4%)
IVA	35 (24.0%)
IVB	105 (71.9%)
Other	1 (0.7%)
Site of metastases^a^	
Brain	90 (61.6%)
Bone	68 (46.6%)
Lung	64 (43.8%)
Thoracic cavity	46 (31.5%)
Pleural	38 (26.0%)
Liver	26 (17.8%)
Kidney	5 (3.4%)
Other	32 (21.9%)
CNS metastasis	90 (61.6%)
With measurable intracranial lesions^b^	42 (47%)
Prior therapy	
Crizotinib	146 (100%)
Chemotherapy	56 (38.4%)
Radiotherapy	29 (19.9%)
Surgery	17 (11.6%)
Other	37 (25.3%)
BOR to prior crizotinib treatment	
CR	1 (0.7%)
PR	89 (61.0%)
SD	31 (21.2%)
PD	3 (2.1%)
NE	4 (2.7%)
NA	18 (12.3%)
Progression on crizotinib treatment	146 (100%)
Prior brain radiotherapy^b^	
Yes	20 (22%)
No	70 (78%)

The PPS was the same as FAS. Data are presented as mean ± standard deviation or *n* (%)

*FAS* full analysis set, *ECOG* Eastern Cooperative Oncology Group, *PS* performance status, *CNS* central nervous system, *BOR* best objective response, *CR* complete response, *PR* partial response, *SD* stable disease, *PD* progressive disease, *NE* not evaluable, *NA* not available, *PPS* per-protocol set

^a^ Multiple sites of metastases per patient existed

^b^ Percent was based on patients with CNS metastases (*n* = 90)

### Efficacy

**Fig. 2:**
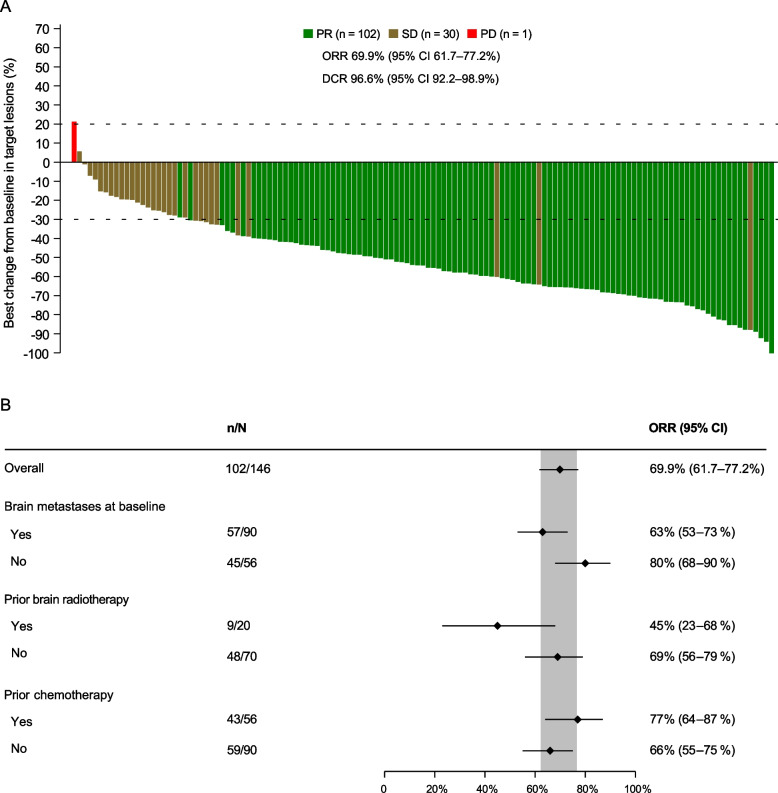
Waterfall plot of systemic BOR (**A**) and forest plot of systemic objective response in subgroups (**B**). Response was assessed by IRC in the FAS (the same as the PPS). The dashed lines at 20% and -30% indicate the thresholds for PD and PR, respectively. There were nine and four patients with IRC-assessed BOR of non-CR/non-PD and NE, respectively. BOR, best objective response; CR, complete response; PR, partial response. SD, stable disease. PD, progressive disease. ORR, objective response rate. DCR, disease control rate. IRC, independent review committee. NE, not evaluable. FAS, full analysis set. PPS, per-protocol set

For all 146 patients in the FAS and PPS, 102 (69.9%) patients had PR, nine (6.2%) patients had non-CR/non-progressive disease (PD), and 30 (20.5%) patients had SD. IRC-assessed ORR was 69.9% (95% CI 61.7–77.2%; Table 2 and Fig. 2A).

**Table 2:** Efficacy of iruplinalkib in FAS

Efficacy	Results (*n* = 146)
IRC-assessed	
BOR	
CR	0
PR	102 (69.9%)
Non-CR/non-PD	9 (6.2%)
SD	30 (20.5%)
PD	1 (0.7%)
NE	4 (2.7%)
Objective response	102 (69.9%; 95% CI 61.7–77.2%)
Disease control	141 (96.6%; 95% CI 92.2–98.9%)
DoR	
Events, *n*/*N* responders (%)	38/102 (37.3%)
Median, months	14.4 (95% CI 13.1–NE)
PFS	
Events	56 (38.4%)
Median, months	19.8 (95% CI 14.5–NE)
TTP	
Events	54 (37.0%)
Median, months	NE (95% CI 14.5–NE)
Investigator-assessed	
Intracranial CR in patients with measurable intracranial lesions at baseline	3/42 (7%; 95% CI 1–19%)
Intracranial objective response in patients with CNS metastases at baseline	41/90 (46%; 95% CI 35–56%)
Intracranial objective response in patients with measurable intracranial lesions at baseline	27/42 (64%; 95% CI 48–78%)
12-month OS rate	85.2% (95% CI 78.2–90.1%)
24-month OS rate	57.9% (95% CI 44.2–69.4%)

The PPS was the same as FAS. Data are presented as *n* (%) or *n*/*N* (%; 95% CI) unless otherwise specified. For patients with systemic CR or PR, response confirmation was required. For patients with intracranial CR or PR, response confirmation was not required. OS data were immature, with 106 (72.6%) censored

*FAS* full analysis set, *PPS* per-protocol set, *IRC* independent review center, *BOR* best objective response, *CR* complete response, *PR* partial response, *PD* progressive disease, *SD* stable disease, *NE* not evaluable, *DoR* duration of response, *PFS* progression-free survival, *TTP* time to progression, *CI* confidence interval, *OS* overall survival, *CNS* central nervous system

The corresponding lower limit of the 95% CI was superior to the pre-defined threshold 40%. Thus, the primary endpoint was met. The benefits were considerable, regardless of baseline brain metastases, previous brain radiotherapy, and previous chemotherapy or not. Systemic ORRs by IRC of patients without and with brain metastases were 80% (95% CI 68–90%) and 63% (95% CI 53–73%), respectively. The systemic ORR of patients not receiving prior brain radiotherapy and that of those receiving prior brain radiotherapy were 69% (95% CI 56–79%) and 45% (95% CI 23–68%), respectively. The forest plot and waterfall plots of the systemic best objective response by IRC in subgroups were shown in Fig. 2B and Additional file 1: Fig. S1, respectively. The DCR by IRC was 96.6% (95% CI 92.2–98.9%). Investigator-assessed ORR and DCR were 63.0% (95% CI 54.6–70.8%) and 94.5% (95% CI 89.5–97.6%), respectively (Additional file 1: Table S2 and Fig. S2). In the RES, IRC-assessed ORR was 74.5% (95% CI 66.3–81.5%) and DCR was 96.4% (95% CI 91.7–98.8%).

**Fig. 3:**
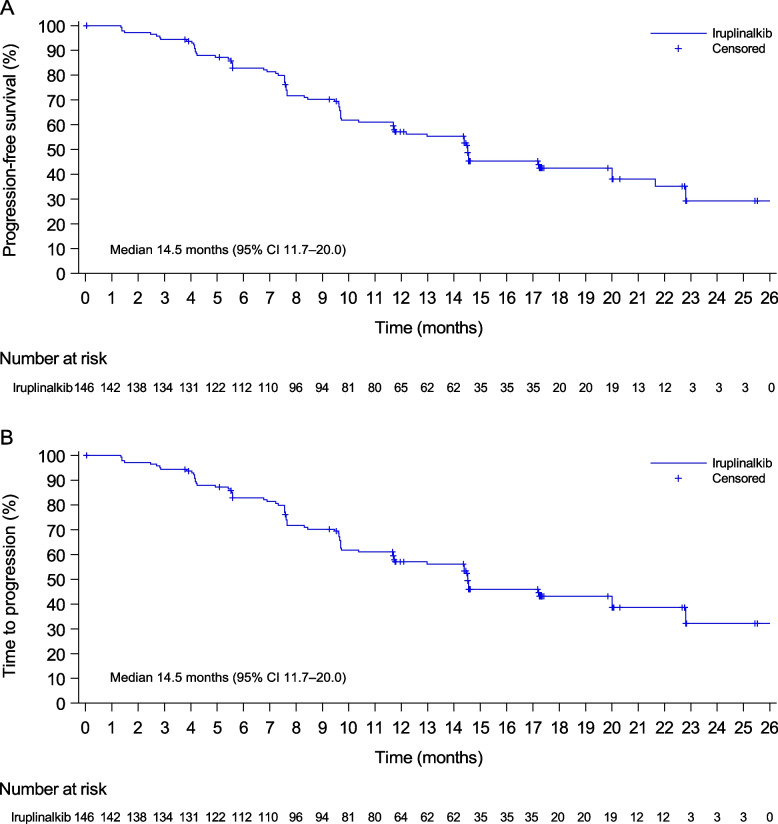
Kaplan-Meier plots of progression-free survival (**A**) and time to progression (**B**) by investigator. Data were from the full analysis set (the same as the per-protocol set). CI, confidence interval

Investigator-assessed median DoR was 13.2 months (95% CI 10.4–17.7; Additional file 1: Table S2 and Fig. S3). Investigator-assessed median PFS was 14.5 months (95% CI 11.7–20.0; Fig. 3A, Additional file 1: Table S2 and Fig. S3). Investigator-assessed median TTP was the same as investigator-assessed median PFS (Fig. 3B, Additional file 1: Table S2 and Fig. S3). IRC-assessed median DoR, PFS, and TTP were 14.4 months (95% CI 13.1-not evaluable [NE]), 19.8 months (95% CI 14.5–NE), and NE (95% CI 14.5–NE), respectively (Table 2).

Among 90 patients with CNS metastasis, the iORR was 46% (41/90, 95% CI 35–56%). For 42 patients with measurable intracranial lesions, iORR was 64% (27/42, 95% CI 48–78%) (Table 2).

At the data cut-off date on November 30, 2021, OS data were immature, with 106 (72.6%) patients censored. The 12-month and 24-month survival rates were estimated to be 85.2% (95% CI 78.2–90.1%) and 57.9% (95% CI 44.2–69.4%), respectively (Table 2).

### Safety

All 146 patients were included in the SS, and 136 (93.2%) patients had TRAEs. The most common TRAEs were AST increased (63 [43.2%]), ALT increased (54 [37.0%]), blood creatine phosphokinase increased (51 [34.9%]), hypercholesterolemia (49 [33.6%]), hypertriglyceridemia (39 [26.7%]), and hypertension (26 [17.8%]). Grade 3-4 TRAEs occurred in 45 (30.8%) patients. Twenty-one (14.4%) and 16 (11.0%) patients suffered TRAEs leading to dose interruption and reduction of iruplinalkib, respectively. The most common reasons for dose reduction of iruplinalkib were AST increased (four [2.7%]), blood creatine phosphokinase increased, rash (three [2.1%], each), gamma-glutamyltransferase increased, and eczema (two [1.4%], each). Dose discontinuations of iruplinalkib due to TRAEs were reported in four (2.7%) patients (blood creatinine increased, interstitial lung disease, rash, and hemorrhage intracranial [0.7%, each]; Table 3). Three patients are recovering from their respective TRAE after discontinuing iruplinalkib, while one patient died of TRAE (hemorrhage intracranial). Additionally, two (1.4%) patients had pneumonitis, both of which were grade 2 and occurred on the 7th day after iruplinalkib treatment initiation, leading to one patient of dose interruption and one patient of dose reduction, respectively. Pneumonitis did not recur after the iruplinalkib treatment continuation.

**Table 3:** TRAEs of iruplinalkib in SS

TRAEs	Patients (*n* = 146)
Any grade TRAEs	136 (93.2%)
Grade 3–4 TRAEs	45 (30.8%)
Serious TRAEs	10 (6.8%)
TRAEs leading to dose interruption	21 (14.4%)
TRAEs leading to dose reduction	16 (11.0%)
TRAEs leading to dose discontinuation	4 (2.7%)
TRAEs in > 10% patients	
AST increased	63 (43.2%)
ALT increased	54 (37.0%)
Blood creatine phosphokinase increased	51 (34.9%)
Hypercholesterolemia	49 (33.6%)
Hypertriglyceridemia	39 (26.7%)
Hypertension	26 (17.8%)
Blood creatine phosphokinase MB increased	24 (16.4%)
Vomiting	23 (15.8%)
Nausea	20 (13.7%)
γ-GT increased	19 (13.0%)
Rash	19 (13.0%)
Blood lactate dehydrogenase increased	19 (13.0%)

Data are presented as *n* (%). One death of hemorrhage intracranial was considered treatment-related

*SS* safety set, *TRAEs* treatment-related adverse events, *AST* aspartate aminotransferase, *ALT* alanine aminotransferase, *γ-GT* gamma-glutamyltransferase

### PK analysis

PK data were available in 141 patients. Plasma iruplinalkib concentration reached a steady state at Cycle 1 Day 21 with a mean C_min,ss_ of 255.4 ± 160.8 ng/mL. C_min,ss_ at each time point were detailed in Additional file 1: Fig. S4.

## Discussion

This phase II study showed that iruplinalkib (WX-0593) had favorable clinical efficacy and manageable safety profiles in patients with *ALK*-positive crizotinib-resistant advanced NSCLC.

Iruplinalkib achieved the primary endpoint with an IRC-assessed ORR of 69.9% (95% CI 61.7–77.2%) whose lower limit of the 95% CI exceeded the pre-defined threshold of 40%. In this study, investigator-assessed ORR was 63.0% (95% CI 54.6–70.8%), consistent with data from the phase I study of iruplinalkib with an investigator-assessed ORR of 63.0% (95% CI 47.5–76.8%) in the dose-escalation phase and 58.2% (95% CI 47.4–68.5%) in the dose-expansion phase [13]. In crizotinib-resistant patients, the proportions of patients achieving IRC-assessed objective response for other second-generation ALK TKIs were 39.1% for ceritinib [8], 50% for alectinib [9], 56% for brigatinib [10], 52% for ensartinib [11], 33.3% for conteltinib [12], and 73% for lorlatinib [15]. Iruplinalkib also showed a DCR of 96.6% (95% CI 92.2–98.9%) assessed by IRC and 94.5% (95% CI 89.5–97.6%) by investigator.

Subgroup analysis of ORR in previous clinical studies was limited. Studies of alectinib and brigatinib reported the results in patients with or without previous chemotherapy. In patients with previous chemotherapy, ORRs were 44% for alectinib [9], 54% for brigatinib [16], and 77% for iruplinalkib in this study. In patients not receiving previous chemotherapy, ORRs were 69%, 52%, and 66% for alectinib, brigatinib, and iruplinalkib, respectively. Additionally, in this study, patients without baseline brain metastases had numerically better systemic ORR compared to those with baseline brain metastases in FAS (80% [95% CI 68–90%] vs 63% [95% CI 53–73%]), patients with prior brain radiotherapy had numerically better ORR compared to those without prior brain radiotherapy in patients with CNS metastatis (69% [95% CI 56–79%] vs 45% [95% CI 23–68%]).

In this study, the median DoR were 14.4 months (95% CI 13.1–NE) by IRC and 13.2 months (95% CI 10.4-17.7) by investigator, while the median DoR for ceritinib was 6.9 months by IRC [8], for alectinib was 11.2 months by IRC [9], for brigatinib 180 mg was 13.8 months by investigator [10], and for conteltinib was 6.6 months by investigator [12]. In respect of median PFS, iruplinalkib had 14.5 months (95% CI 11.7–20.0) by investigator and 19.8 months (95% CI 14.5–NE) by IRC in this study. The median PFS for ceritinib was 5.4 months by IRC and 6.7 months by investigator [8], for alectinib was 8.9 months by IRC [9], for brigatinib 180 mg was 16.7 months by IRC and 15.6 months by investigator [10], for ensartinib was 9.6 months by investigator [11], and for conteltinib was 6.7 months by investigator [12]. In the dose-expansion phase of the phase I study of iruplinalkib, because of the short median follow-up period of 203 days (95% CI 194–219), PFS data were immature [13].

Several studies suggested that CNS progression occurred frequently within one year during crizotinib treatment [7]. Second-generation ALK TKIs showed good intracranial antitumor activity. In this study, iruplinalkib demonstrated promising intracranial efficacy, with an iORR of 64% in patients with intracranial measurable lesions at baseline. That for alectinib was 57% [9]. And that for brigatinib 180 mg was 67% [10]. For conteltinib, iORR was 33.3% in six patients with measurable intracranial lesions [12]. Intracranial CR rate was 7% for iruplinalkib, numerically similar to ceritinib (8.7%) [17], alectinib (8.3%) [18], and brigatinib 90 mg (8%) [10]. However, no intracranial CR was reported in the brigatinib 180 mg [10]. These results suggested that iruplinalkib penetrates the blood-brain barrier and is an option for *ALK*-positive advanced NSCLC patients with brain metastases, similar to the other second-generation ALK TKIs.

In this study, OS data were immature, with a median follow-up of 18.2 months (95% CI 16.8–18.8). Estimated 12-month and 24-month OS rates were 85.2% (95% CI 78.2–90.1%) and 57.9% (95% CI 44.2–69.4%), respectively. The 12-month OS rates for alectinib and brigatinib 180 mg, and 24-month OS rate for brigatinib 180 mg were 71% [19], 80%, and 66% [10], respectively.

In terms of RP2D, in the dose-expansion phase of the previous iruplinalkib phase I study, two patients who received iruplinalkib 120 mg orally once daily without the lead-in dose died due to infectious pneumonia and respiratory failure, which occurred within 48 h after iruplinalkib treatment initiation, respectively [13]. Brigatinib without a lead-in phase may increase the risk of early-onset pulmonary events (EOPEs). And lead-in phase was implemented to lower the risk [20]. Although whether iruplinalkib could result in EOPEs was unconfirmed, dosing cohorts of 120 mg and 180 mg with a 7-day lead-in phase at 60 mg orally once daily were added to the iruplinalkib phase I study for safety concerns. And finally, iruplinalkib 180 mg orally once daily with a 7-day lead-in phase at 60 mg orally once daily was determined as the RP2D [13]. Further studies are needed to determine the mechanisms behind these AEs.

For safety, TRAEs for iruplinalkib occurred in 93.2% patients, with a numerically lower incidence of anemia, constipation, and rash than alectinib [9], brigatinib [10], and ensartinib [11], a numerically lower incidence of visual disturbances, diarrhea, edema, and renal impairment compared to alectinib [19], and a numerically lower incidence of elevated blood creatinine and fatigue compared to ensartinib [11]. Hypercholesterolemia and hypertriglyceridemia were also common with iruplinalkib, and it is unclear how dysregulation of lipid metabolism occurs. The majority of TRAEs in this study were grade 1 or 2. Dose interruption, reduction, and discontinuation due to TRAEs occurred in 14.4%, 11.0%, and 2.7% of patients receiving iruplinalkib, respectively. Dose discontinuation rate for alectinib was 8% [9]. Dose interruption, reduction, and discontinuation rates were 62%, 29%, and 11% for brigatinib 180 mg [10], 15%, 12%, and 3% for ensartinib [11], and 6.3%, 4.7%, and 3.1% for conteltinib [12], respectively. The incidences of grade 3-4 and serious TRAEs for iruplinalkib (30.8% and 6.8%) were numerically comparable to those for ensartinib (23% and 8%) [11]. The safety profiles of iruplinalkib reported in this study were generally consistent with that in the 180 mg group of the previous iruplinalkib phase I study (TRAEs: 95%; Grade ≥ 3 TRAEs: 23%; Serious TRAEs: 8%; Dose interruption, reduction, and discontinuation due to TRAEs: 18%, 3%, and 5%, respectively) [13]. Further, pneumonitis occurred in 1.4% patients in the present study. In 120 mg or 180 mg with or without lead-in phase in the iruplinalkib phase I study, pneumonitis (2%) only occurred in the 180 mg without lead-in phase. One treatment-related death of hemorrhage intracranial was reported. Together, iruplinalkib has manageable safety profiles. However, when brain metastatic patients with high cardiovascular disease risks receive iruplinalkib, special attention should be paid to hemorrhage intracranial.

For PK analysis, mean C_min,ss_ of iruplinalkib were 255.4 ± 160.8 ng/mL and 164.10 ± 77.76 ng/mL in the present study and phase I study [13], respectively. Large standard deviations of drug concentration of iruplinalkib indicated differences in individuals. Future population PK studies could assess the factors that influence PK of iruplinalkib.

This study has some limitations. The OS data were immature because of insufficient follow-up duration. Updated OS data were awaited. There was also a lack of detection of efficacy predictors before and resistance mutations after iruplinalkib treatment. These could be investigated in the further studies. Besides, this study only enrolled Chinese patients, thus caution must be paid when these results were interpreted for other ethnic patients.

## Conclusions

This study demonstrates that iruplinalkib (WX-0593) had favorable efficacy and manageable safety profiles in patients with *ALK*-positive crizotinib-resistant advanced NSCLC. Iruplinalkib could be a new treatment option for this patient population. A phase III study to compare iruplinalkib and crizotinib in the first-line setting (NCT04632758) in *ALK*-positive NSCLC is ongoing.

## Supplementary Information

**Additional file 1: Table S1.** Summary of comorbidities in ≥ 5% patients in FAS. **Table S2.** Investigator-assessed efficacy of iruplinalkib in FAS. **Figure S1.** Waterfall plots of the subgroup BOR of target lesions by IRC in FAS. **Figure S2.** Waterfall plot of BOR by investigator in FAS. **Figure S3.** Swimmer plot of iruplinalkib exposure and response by investigator in FAS. **Figure S4.** Minimum plasma iruplinalkib concentration at each time point in the pharmacokinetics analysis set.

## Data Availability

The datasets generated and analyzed during the current study cannot be shared. Reason: We may further analyze the raw data for other research objectives.

## Abbreviations

AE: Adverse event
ALK: Anaplastic lymphoma kinase
ALT: Alanine aminotransferase
AST: Aspartate aminotransferase
CI: Confidence interval
C_min,ss_: Minimum concentration at steady-state
CNS: Central nervous system
CR: Complete response
CT: Computed tomography
CTCAE: Common Terminology Criteria for Adverse Events
DCR: Disease control rate
DoR: Duration of response
ECOG: Eastern Cooperative Oncology Group
EOPE: Early-onset pulmonary event
FAS: Full analysis set
iORR: Intracranial objecctive response rate
IRC: Independent review committee
NE: Not evaluable
NSCLC: Non-small cell lung cancer
ORR: Objective response rate
OS: Overall survival
PCR: Polymerase chain reaction
PD: Progressive disease
PFS: Progression-free survival
PK: Pharmacokinetics
PPS: Per-protocol set
PR: Partial response
PS: Performance status
RANO: Response Assessment in Neuro-Oncology
RECIST: Response Evaluation Criteria in Solid Tumors
RES: Response evaluable set
ROS1: c-ros oncogene 1
RP2D: Recommended phase 2 dose
SD: Stable disease
SS: Safety set
TKI: Tyrosine kinase inhibitor
TRAE: Treatment-related adverse event
TTP: Time to progression
ULN: Upper limit of normal
