# Supplementary material for: Efficacy and safety of iruplinalkib (WX-0593) in ALK-positive crizotinib-resistant advanced non-small cell lung cancer patients: a single-arm, multicenter phase II study (INTELLECT)
Source: BMC Med. 2023 Feb 24;21:72. doi: 10.1186/s12916-023-02738-5 (PMC9960473; doi:10.1186/s12916-023-02738-5)
Supplement: Supplementary file 1 — Additional file 1: Table S1. Summary of comorbidities in ≥ 5% patients in FAS. Table S2. Investigator-assessed efficacy of iruplinalkib in FAS. Figure S1. Waterfall plots of the subgroup BOR of target lesions by IRC in FAS. Figure S2. Waterfall plot of BOR by investigator in FAS. Figure S3. Swimmer plot of iruplinalkib exposure and response by investigator in FAS. Figure S4. Minimum plasma iruplinalkib concentration at each time point in the pharmacokinetics analysis set. [file 12916_2023_2738_MOESM1_ESM.docx]

***Supplementary Material***

**Table S1.** Comorbidities in ≥ 5% patients in FAS

| Comorbidity | Patients (n = 146) |
| --- | --- |
| Hypertension | 23 (15.8%) |
| Renal cyst | 12 (8.2%) |
| Hepatic steatosis | 11 (7.5%) |
| Hypoalbuminaemia | 11 (7.5%) |
| Anaemia | 11 (7.5%) |
| Sinusitis | 10 (6.8%) |
| Cough | 10 (6.8%) |
| Hepatic cyst | 9 (6.2%) |
| Oedema peripheral | 9 (6.2%) |
| Pneumonitis | 8 (5.5%) |
| Sinus bradycardia | 8 (5.5%) |
| Uterine leiomyoma | 8 (5.5%) |

Note: The PPS was the same as the FAS. Data are presented as n (%).

FAS, full analysis set. PPS, per-protocol set.

**Table S2.** Investigator-assessed efficacy of iruplinalkib in FAS

| Efficacy | Patients (n = 146) |
| --- | --- |
| BOR |  |
| CR | 0 |
| PR | 92 (63.0%) |
| Non-CR/non-PD | Not available |
| SD | 46 (31.5%) |
| PD | 4 (2.7%) |
| NE | 4 (2.7%) |
| Objective response | 92 (63.0%; 95% CI 54.6–70.8%) |
| Disease control | 138 (94.5%; 95% CI 89.5–97.6%) |
| DoR |  |
| Events, n/N responders (%) | 48/92 (52%) |
| Median, months | 13.2 (95% CI 10.4–17.7) |
| PFS |  |
| Events | 76 (52.1%) |
| Median, months | 14.5 (95% CI 11.7–20.0) |
| TTP |  |
| Events | 74 (50.7%) |
| Median, months | 14.5 (95% CI 11.7–20.0) |

Note: The PPS was the same as the FAS. Data are presented as n (%) unless otherwise specified. For patients with systemic CR or PR, response confirmation was required. For patients with intracranial CR or PR, response confirmation was not required.

FAS, full analysis set. PPS, per-protocol set. BOR, best objective response. CR, complete response. PR, partial response. PD, progressive disease. SD, stable disease. DoR, duration of response. PFS, progression-free survival. TTP, time to progression. CI, confidence interval.


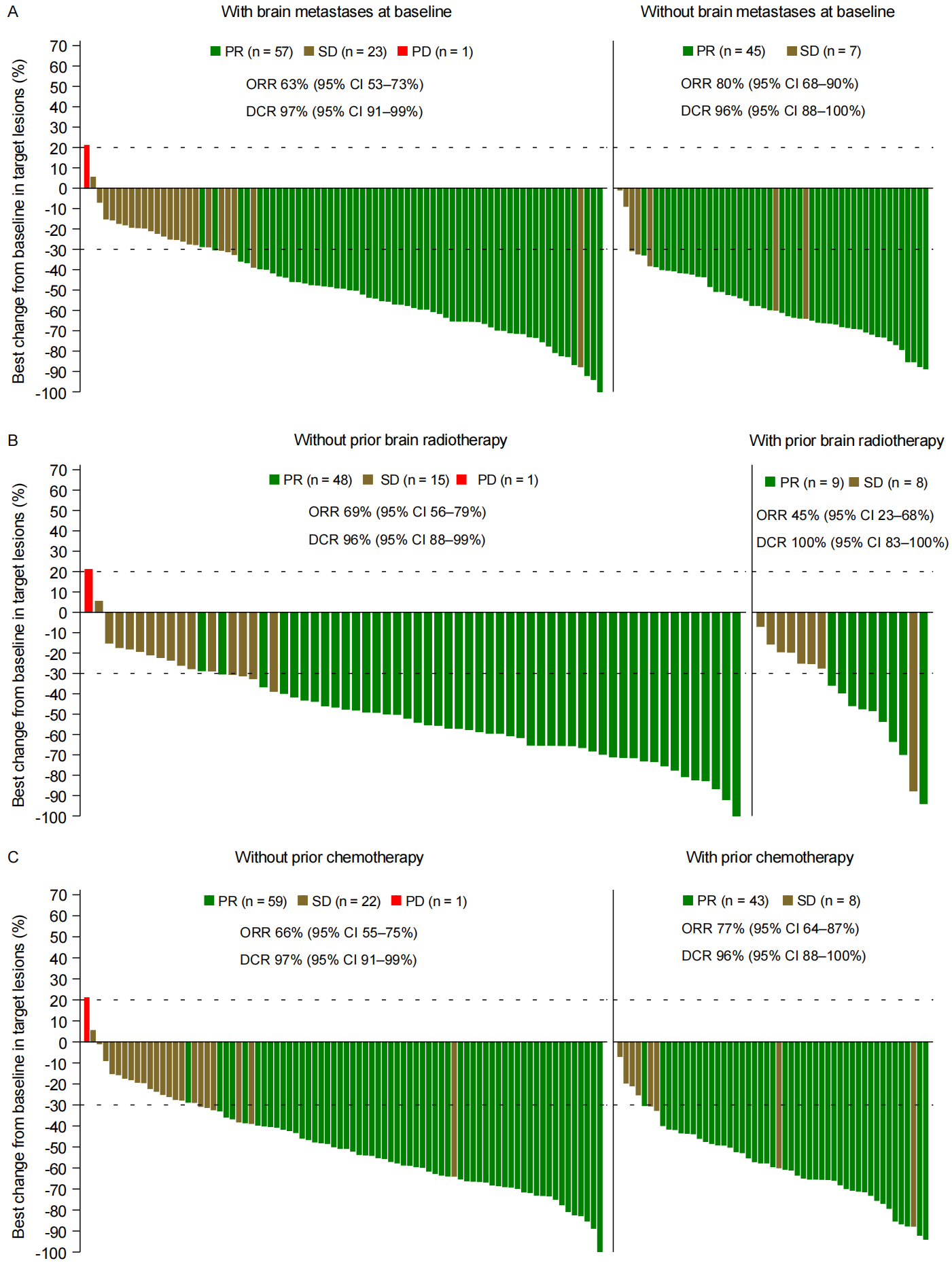


**Figure S1.** Waterfall plots of the subgroup systemic BOR of target lesions by IRC in FAS

(**A**) Patients with brain metastases at baseline or not. Seven non-CR/non-PD and two NE in patients with brain metastases at baseline. Two non-CR/non-PD and two NE in patients without brain metastases at baseline. (**B**) Patients with prior brain radiotherapy or not. Four non-CR/non-PD and two NE in patients without prior brain radiotherapy. Three non-CR/non-PD and zero NE in patients with prior brain radiotherapy. (**C**) Patients with prior chemotherapy or not. Six non-CR/non-PD and two NE in patients without prior chemotherapy. Three non-CR/non-PD and two NE in patients with prior chemotherapy.

Note: The PPS was the same as FAS. The dashed lines at 20% and -30% indicate the thresholds for PD and PR, respectively. PR, partial response. SD, stable disease. PD, progressive disease. ORR, objective response rate. DCR, disease control rate. BOR, best objective response. IRC, independent review committee. FAS, full analysis set. PPS, per-protocol set. NE, not evaluable.


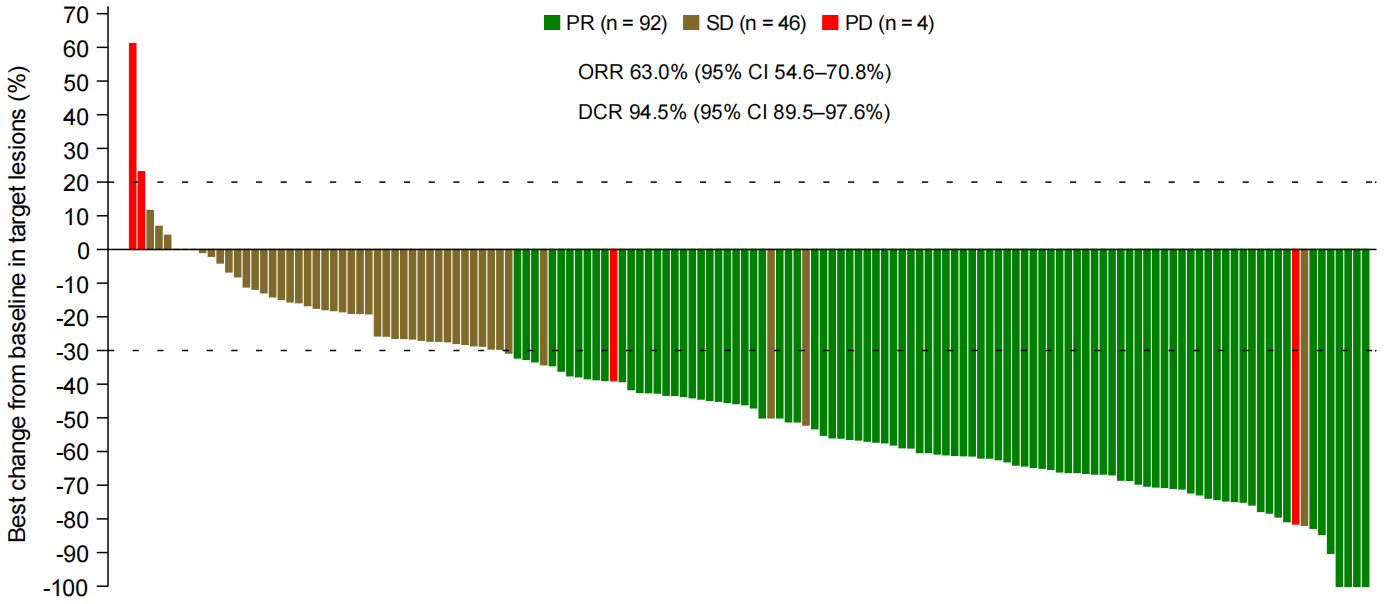


**Figure S2.** Waterfall plot of BOR by investigator in FAS

Note: The PPS was the same as FAS. The dashed lines at 20% and -30% indicate the thresholds for PD and PR, respectively. There were four patients with investigator-assessed BOR of NE. PR, partial response. SD, stable disease. PD, progressive disease. ORR, objective response rate. DCR, disease control rate. BOR, best objective response. FAS, full analysis set. PPS, per-protocol set. NE, not evaluable.


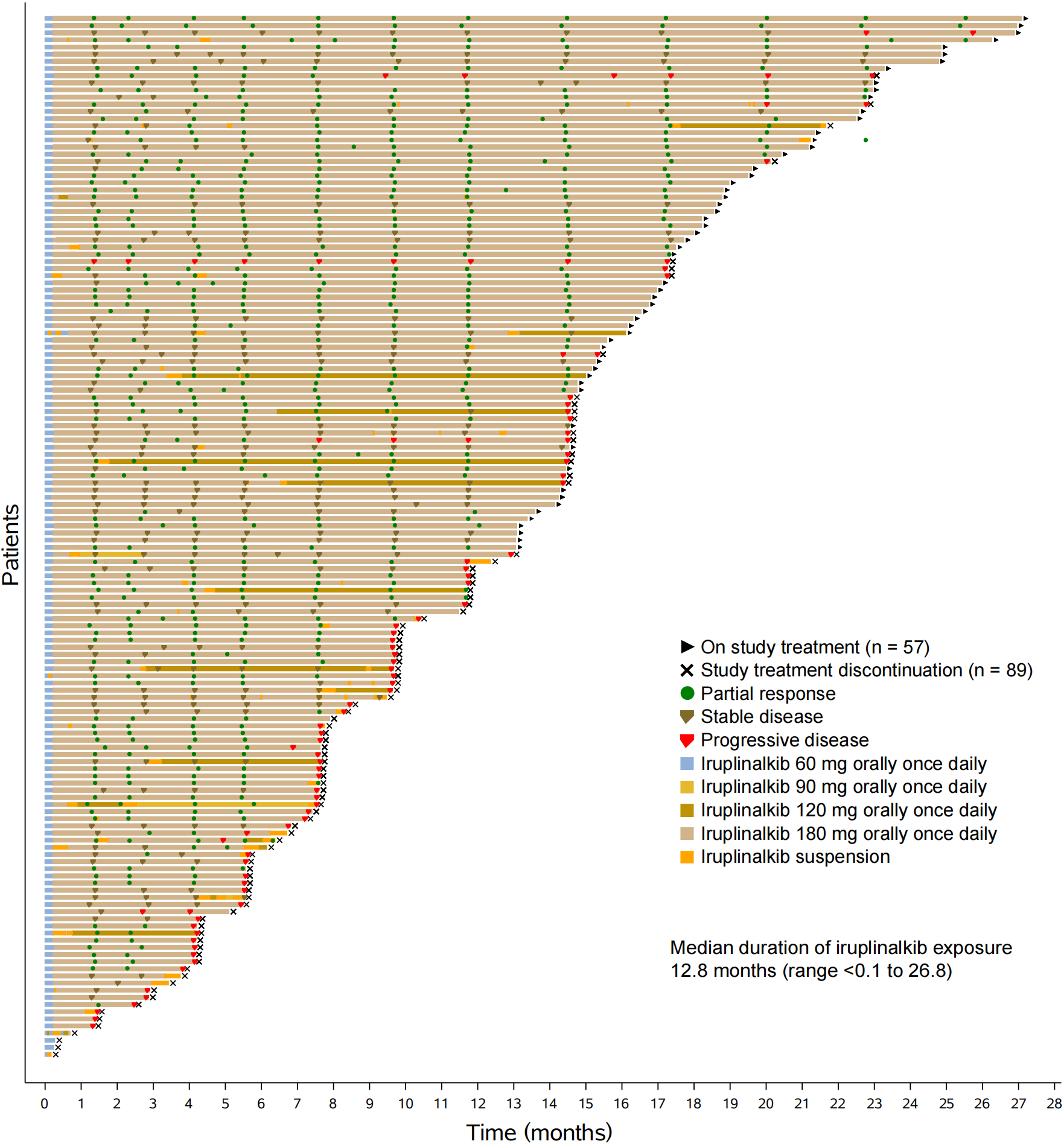


**Figure S3.** Swimmer plot of iruplinalkib exposure and response by investigator in FAS

Note: The PPS was the same as FAS. FAS, full analysis set. PPS, per-protocol set.


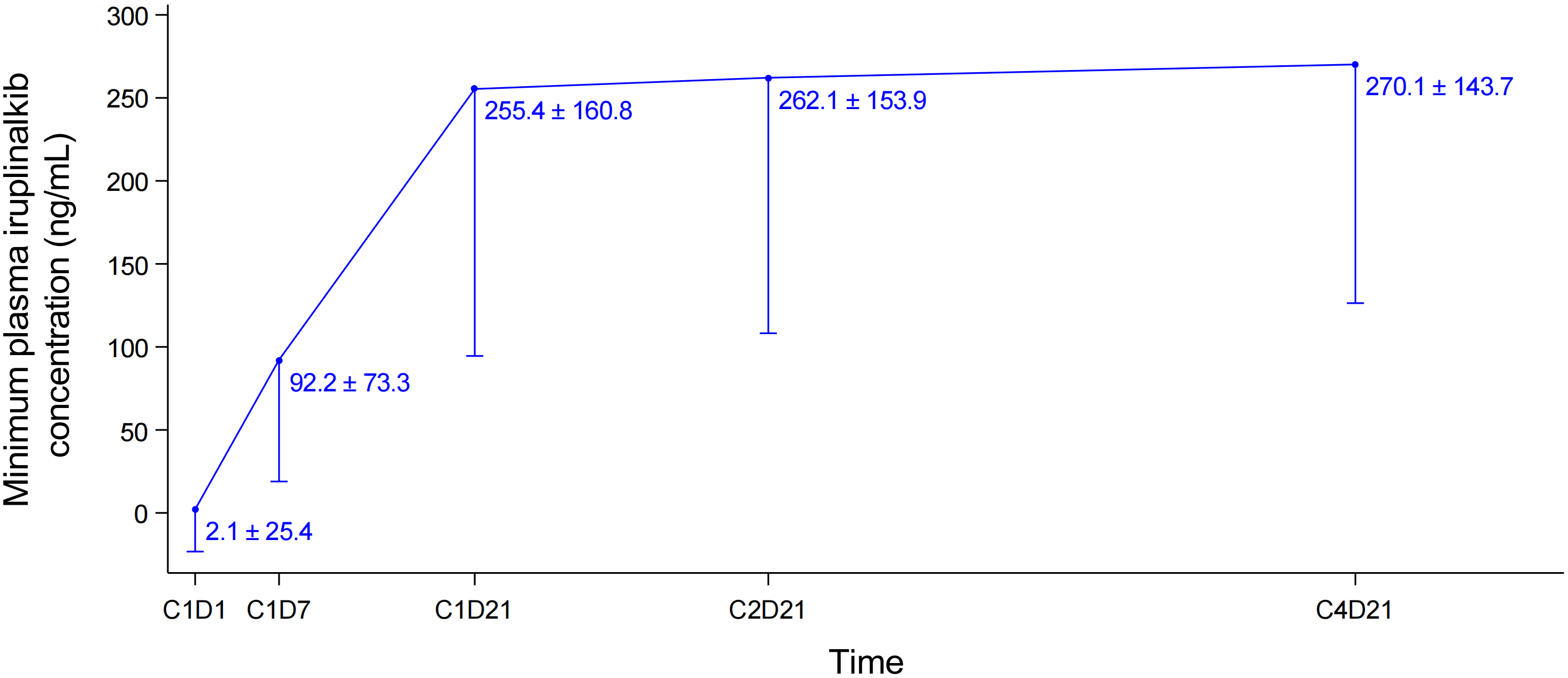


**Figure S4.** Minimum plasma iruplinalkib concentration at each time point in the pharmacokinetics analysis set

Concentration reached steady state at C1D21 (21 days as a cycle).

Note: C1D1, Cycle 1 Day 1. C1D7, Cycle 1 Day 7. C1D21, Cycle 1 Day 21. C2D21, Cycle 2 Day 21. C4D21, Cycle 4 Day 21.
